# Influence of Synthesis Parameters on Structure and Characteristics of the Graphene Grown Using PECVD on Sapphire Substrate

**DOI:** 10.3390/nano14201635

**Published:** 2024-10-12

**Authors:** Šarūnas Jankauskas, Šarūnas Meškinis, Nerija Žurauskienė, Asta Guobienė

**Affiliations:** 1 Institute of Materials Science, Kaunas University of Technology, K. Baršausko St. 59, LT-51423 Kaunas, Lithuania; sarunas.jankauskas@ktu.lt (Š.J.); sarunas.meskinis@ktu.lt (Š.M.); 2 Department of Functional Materials and Electronics, Center for Physical Sciences and Technology, Saulėtekio av. 3, LT-10257 Vilnius, Lithuania; nerija.zurauskiene@ftmc.lt

**Keywords:** graphene, PECVD, sapphire, sheet resistance

## Abstract

The high surface area and transfer-less growth of graphene on dielectric materials is still a challenge in the production of novel sensing devices. We demonstrate a novel approach to graphene synthesis on a C-plane sapphire substrate, involving the microwave plasma-enhanced chemical vapor deposition (MW-PECVD) technique. The decomposition of methane, which is used as a precursor gas, is achieved without the need for remote plasma. Raman spectroscopy, atomic force microscopy and resistance characteristic measurements were performed to investigate the potential of graphene for use in sensing applications. We show that the thickness and quality of graphene film greatly depend on the CH_4_/H_2_ flow ratio, as well as on chamber pressure during the synthesis. By varying these parameters, the intensity ratio of Raman D and G bands of graphene varied between ~1 and ~4, while the 2D to G band intensity ratio was found to be 0.05–0.5. Boundary defects are the most prominent defect type in PECVD graphene, giving it a grainy texture. Despite this, the samples exhibited sheet resistance values as low as 1.87 kΩ/□. This reveals great potential for PECVD methods and could contribute toward efficient and straightforward graphene growth on various substrates.

## 1. Introduction

High charge carrier mobility, mechanical strength and optical transparency are the properties that make graphene standout from other materials developed for high-speed electronics [1], solar cell [2] and sensor [3] applications. For example, the graphene/dielectric interface shows a lot of promise for sensing devices. Temperature sensors based on the graphene/SiN configuration perform excellently and have a very fast response and mechanical stability [4]. Graphene can also be used in magnetoresistance sensors, which are optimal in the 0.1–20 T range, when grown on polycrystalline Al_2_O_3_ substrates [5]. The peculiar nature of graphene in humidity and carbon dioxide sensing was also investigated when SiO_2_ and sapphire substrates were selected [6]. It has been shown that device performance also relies on the substrate used, not only the quality of the graphene. Despite this, most concepts utilize exfoliated graphene, due to its quality (low defect density) [3,7,8]. However, exfoliation fails when large-area deposition is required [9,10]. Chemical vapor deposition (CVD) synthesis methods are far superior when it comes to graphene scalability [11,12,13]. To maintain the pristine quality of graphene in terms of defect density and domain (flake) size, most CVD methods focus on synthesis that requires Cu or other types of metal catalyst films [14,15,16]. The extra transfer step has to be taken into account for the proper utilization of graphene on desired substrates. The transfer of graphene from catalytic surfaces onto various substrates is quite complicated [17,18] and usually results in unwanted contamination, cracking or even the destruction of the initial graphene film [19,20]. Changing the growth parameters and experimenting with different substrates might produce better results. For example, it was shown that graphene can be grown directly on SiC, with high charge carrier mobility and the ability to tune graphene properties by varying the doping amounts [21]. Similarly, there have been attempts to synthesize graphene on a Si(100) and SiO_2_ substrate directly, using plasma-enhanced chemical vapor deposition (PECVD) [22,23,24]. In this way, graphene synthesis temperatures were significantly reduced (below 700 °C). However, directly synthesized graphene is nanocrystalline, and boundary defects dominate such samples [25,26].

During the last decade, there have been attempts to synthesize graphene on Al_2_O_3_ substrates [27,28,29,30,31], and particular attention was given to C-plane sapphire substrates [32,33,34]. Having a catalytic nature, C-plane sapphire ensures a more stable growth process, eliminates the unwanted grain boundary effects and significantly increases the size of graphene crystallites, due to the naturally matched hexagonal graphene and sapphire structures [35]. Recent reports indicate the enormous potential of graphene/C-plane sapphire interface. Wafer-scale, highly oriented graphene, exhibiting high carrier mobility and low sheet resistance, was directly grown on sapphire [36]. Sapphire is also an excellent substrate for GaN remote epitaxy assisted by a graphene interlayer; in this case, directly grown graphene is more beneficial than transferred graphene [37]. It has been recently shown that graphene synthesis can also be activated using Al-enriched C-plane sapphire substrates [38]. However, high quality films have only been produced using CVD, where temperatures well above 1000 °C are required [36]. The use of the PECVD would allow us to substantially decrease the temperature of synthesis. However, only a handful of studies have been performed on direct catalyst-less graphene growth on sapphire substrate using PECVD [39,40,41]. The use of PECVD provides an additional opportunity to control the graphene synthesis conditions. However, in the case of graphene PECVD synthesis on sapphire, only the effects of hydrogen and acetylene gas flows on the graphene structure were studied [39].

In this research, the effects of the graphene synthesis pressure and the ratio of hydrogen to methane gas flows were investigated. We show a way to couple both substrate catalytic effects and low-temperature synthesis mechanisms to make graphene growth on C-plane sapphire substrates more efficient and cost-effective. The small chamber size and the ability to change the synthesis conditions in a wide range of parameters provide versatility for further industrial development prospects. Paired with the ability to produce fairly low-resistance graphene films, this method could be considered as a viable option for sensing applications.

## 2. Materials and Methods

**Figure 1:**
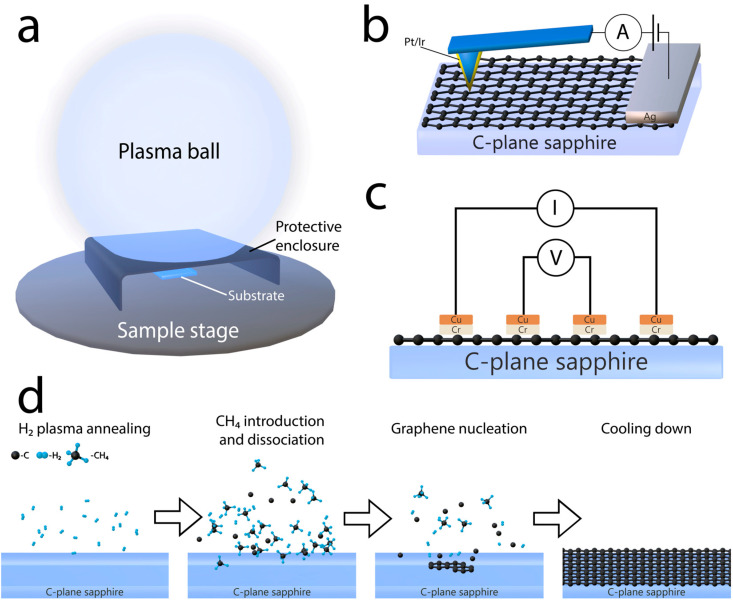
(**a**) Schematic of the PECVD chamber during graphene synthesis on C-sapphire substrate, (**b**) illustration of C-AFM analysis, (**c**) illustration of four-point probe analysis for sheet resistance measurement and (**d**) schematic representation of PECVD graphene synthesis stages. Atom spacing, electrode placement and dimensions are not to scale for clarity.

The synthesis of graphene/C-plane sapphire samples was performed using a microwave PECVD system, Cyrannus (Innovative Plasma Systems (Iplas) GmbH, Troisdorf, Germany). Monocrystalline, double-side-polished, 10 × 10 × 0.5 mm C-plane (0001) sapphire (AdValue Technology, Tuscon, AZ, USA) was used as a substrate for all samples (Figure 1a). The synthesis was carried out in a similar way to our previous work [42,43]. A schematic of our growth stages is depicted in Figure 1d. A mixture of hydrogen and methane was used as the precursor gas in all cases. Initially, plasma was ignited using hydrogen gas until the target temperature was reached. After that, a 10 min. H_2_ plasma annealing phase followed , where the flow of H_2_ was set to 200 sccm. Hereinafter, the H_2_ flow was reduced, and methane was introduced into the chamber according to the selected growth condition. We produced two separate sample sets. A set, named F, consisted of samples where different CH_4_ gas flows were used, keeping the sum of CH_4_ and H_2_ gas flows at 100 sccm and leaving other parameters fixed. Another set, named P, consisted of samples where the pressure was changed for each synthesis, while maintaining the CH_4_/H_2_ gas-flow ratio (35/65 sccm) and keeping the other parameters fixed. Additionally, three more samples were synthesized in order to populate the gas-flow ratio and pressure-space colormaps (set named S). The synthesis conditions of all graphene/C-plane sapphire samples can be seen in Table 1. To prevent excessive direct plasma action during each graphene synthesis process, C-plane sapphire substrates were covered with a protective steel enclosure, as seen in Figure 1a.

The graphene structure was analyzed using Raman scattering spectroscopy (Renishaw inVia microscope, Wotton-under-Edge, UK). The excitation wavelength for all measurements was 532 nm, and a beam power of 1.5 mW was used. Five measurements were performed for each sample just after the synthesis of graphene films, to average the structural effects across the sample. For the detection of graphene fingerprint, the main D peak at ~1350 cm^−1^, G peak at ~1600 cm^−1^ and 2D peak at ~2700 cm^−1^ were analyzed and fitted using a Lorentzian function [44]. Defects were analyzed using the D and G peak intensity ratio (I_D_/I_G_) [44,45,46], while the thickness of graphene was determined using the 2D and G peak intensity ratio (I_2D_/I_G_) [47]. Strain and doping were investigated using the position and full-width at half maximum (FWHM) of the peaks [46,48].

The surface conductivity and morphology of the directly synthesized graphene sheets were analyzed by atomic force microscopy (NanoWizard^®^3, JPK Instruments, Bruker Nano GmbH, Berlin, Germany). The morphology images of each sample were collected using an ACTA (Applied NanoStructures, Inc., Mountain View, CA, USA) probe operating in tapping mode. The probe tip radius of curvature was 6 nm. The conductivity was measured using contact-mode conductive atomic force microscopy (C-AFM) with a metal-coated tip ANSCM-PT (AppNano, Mountain View, CA, USA) silicon probe with a thin layer of Pt/Ir coating (thickness (nm) −25 ± 5) on both the reflex and tip sides of the probe. ANSCM probes with a 1.6 spring constant are ideal for use in C-AFM mode (as shown in Figure 1b). Tip shape: tetrahedral; tip ROC (nm): 30; height (μm): 14–16; frequency (kHz): 61. The electrical current was measured as a function of the applied bias voltage (−10–10 mV). All the measurements were performed at room temperature in the air [49].

To test resistive characteristics, samples F2, F5, P1, P5 were selected. A horizontal row of rectangular 2 × 1 mm contacts, which are spaced 1 mm apart, was deposited on the graphene/C-plane sapphire. Electrical contacts were made from Cr and Cu with thicknesses of 20 and 200 nm, respectively. The samples were analyzed with the GOM-805 DC Milli-Ohm Meter (GW Instek, New Taipei City, Taiwan), using the four-point probe method at ambient pressure and room temperature, as shown in Figure 1c. The acquired resistance values were corrected for the geometry and arrangement of the contacts.

## 3. Results

**Figure 2:**
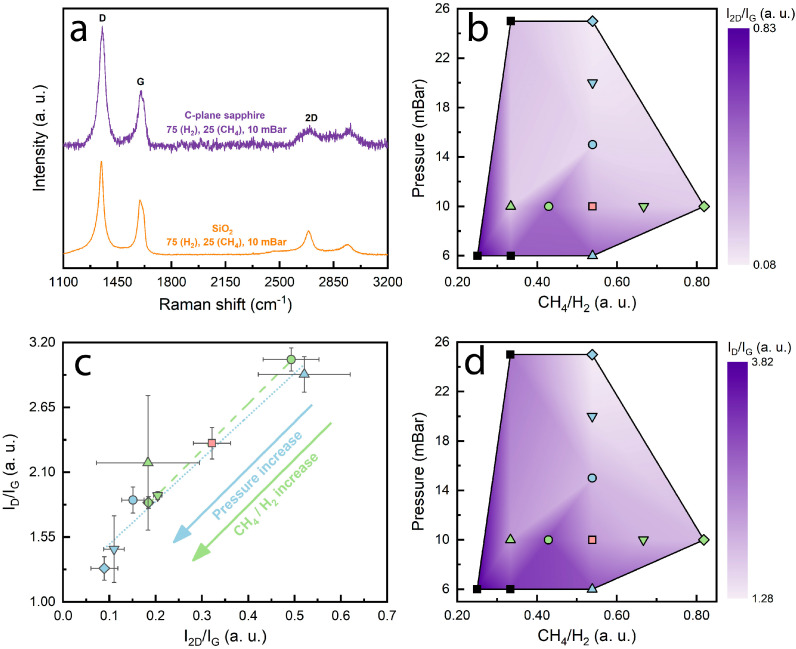
(**a**) Comparison of Raman spectra of PECVD graphene on C−plane sapphire (violet) and SiO_2_ (orange), (**b**) colormap of I_2D_/I_G_ ratio vs. synthesis parameters, (**c**) I_D_/I_G_ ratio vs. I_2D_/I_G_ ratio, (**d**) colormap of I_D_/I_G_ ratio vs. synthesis parameters. The samples were differentiated by shapes and color, where light green represents samples that belong to the F set and light blue represents samples that belong to the P set. Sample F3P2, which belongs to both sets, is colored red. Additional samples were produced (set S) for colormap space expansion.

First, we compare graphene synthesized using MW-PECVD on a C-plane Al_2_O_3_, along with graphene grown on SiO_2_. Most of the samples grown on C-plane sapphire exhibited a wide luminescence background at ~2000 cm^−1^, which had to be subtracted. It was concluded that the substrate was the source of the background in all Raman spectra (Figure S1). The typical, baseline-corrected Raman spectra of the samples grown on C-plane sapphire can be seen in Figure 2a, along with a typical spectrum of graphene synthesized on SiO_2_. Graphene fingerprints are clearly seen, with 2D (~2690 cm^−1^), G (~1595 cm^−1^) and D (~1350 cm^−1^) peaks visible in all spectra. This has also been reported in similar studies, in which graphene was synthesized on Al_2_O_3_ [27,50,51] and SiO_2_ substrates [52,53,54]. An apparent blueshift of the D and G peaks is visible in the graphene grown on C-plane sapphire as compared to the graphene grown on SiO_2_. Furthermore, an in-depth analysis of the graphene synthesized on C-plane sapphire was performed. The thickness of the films can be discovered from the I_2D_/I_G_ relation [55,56]. In our case, when the CH_4_/H_2_ ratio of 35/65 sccm was used and the pressure was set to 6 mBar, I_2D_/I_G_ was 0.68, which indicates a few-layered graphene film as compared to the case of 25 mBar, where I_2D_/I_G_ was 0.09, indicating a multilayer graphene film. A summary of I_2D_/I_G_ and I_D_/I_G_ with respect to different gas-flow ratios and chamber pressures used is shown in Figure 2b,d. The contour plot was interpolated from the points, with two cross sections indicating pressure and precursor gas-flow ratio parameter sweeps (Figure 2b,d: green and blue points, along with the red intersecting point). Increasing pressure ultimately increases I_2D_/I_G_, and a similar increase is observed with increased CH_4_/H_2_ gas flow. We also consider the I_D_/I_G_ ratio, which reveals grain boundary defects [57] (Figure 2d). We show that the number of defects increases with a decrease in pressure and the CH_4_/H_2_ flow ratio, and it correlates linearly with I_2D_/I_G_ (Figure 2c). A large increment in the growth conditions introduced a lot of variability in our reported I_D_/I_G_ and I_2D_/I_G_ values. The distribution of values across a single sample may be associated with the inability to control certain synthesis elements, such as plasma homogeneity and substrate placement in the chamber prior to growth. This relation suggests that graphene grows more disorderly when the graphene thickness is low. As we approach thicker graphene films, the structure of samples tends to be more uniform. This might be attributed to higher amounts of boundary defects, which indicate a rather polycrystalline graphene film, as previously reported [43]. Similar conclusions can be drawn based on Raman theory [58,59]. When observing the I_D_/I_D’_ data with respect to the synthesis conditions used (Figure S2a,b), most of our samples exhibit boundary-like defects associated with the nanocrystalline nature of our samples, where I_D_/I_D’_—~3.5. Only when we drastically increase the gas-flow ratio and pressure, the value of I_D_/I_D’_ reduces to ~2.6, indicating a superposition of boundary and on-site defects. This could occur due to the increase in the byproducts of dissociation, when the pressure and CH_4_ content are higher. When the synthesis pressure is lower, we can see vacancy and boundary defect superposition, which result from the poor nucleation of carbon atoms at the C-plane sapphire surface.

**Figure 3:**
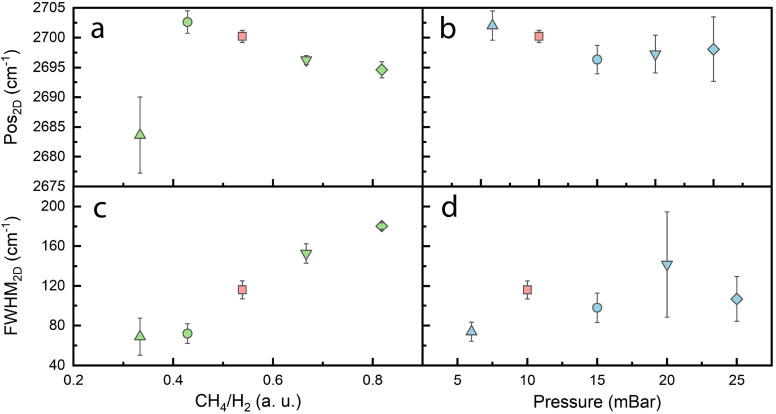
(**a**) Pos_2D_ vs. gas-flow ratio plot, (**b**) Pos_2D_ vs. pressure plot, (**c**) FWHM_2D_ vs. gas-flow ratio plot, (**d**) FWHM_2D_ vs. pressure plot of F and P samples. Conveniently, samples were given the same shapes and colors for distinction.

To assess the effects induced by strain, the 2D peak position and its full width at half maximum (FWHM_2D_) were considered [38,60,61,62] (Figure 3). Although the distribution of the 2D peak position is broad when the CH_4_/H_2_ flow ratio of 25/75 is used, there is a clear decrease in the 2D peak position values (from 2702 cm^−1^ to 2694 cm^−1^) when the precursor gas-flow ratio increases further (Figure 3a). This is in agreement with other reports from similar studies [30,34] and can be attributed to the stress induced in the graphene structure after the deposition process due to the thermal expansion coefficient mismatch [61,62]. FWHM_2D_ is high in all cases, increasing from ~70 cm^−1^ to ~180 cm^−1^ when the CH_4_/H_2_ flow ratio is increased (Figure 3c). Multilayer graphene must be considered, and the change might be directly related to the increase in carbon species during deposition [63,64]. Ultimately, it shows a non-uniform strain distribution in all of our samples [38]. In contrast, when we increase the chamber pressure, the deviation of the 2D peak position increases, while the values remain similar (Figure 3c). There is no correlation between FWHM_2D_ and changes in chamber pressure, as seen in Figure 3d.

**Figure 4:**
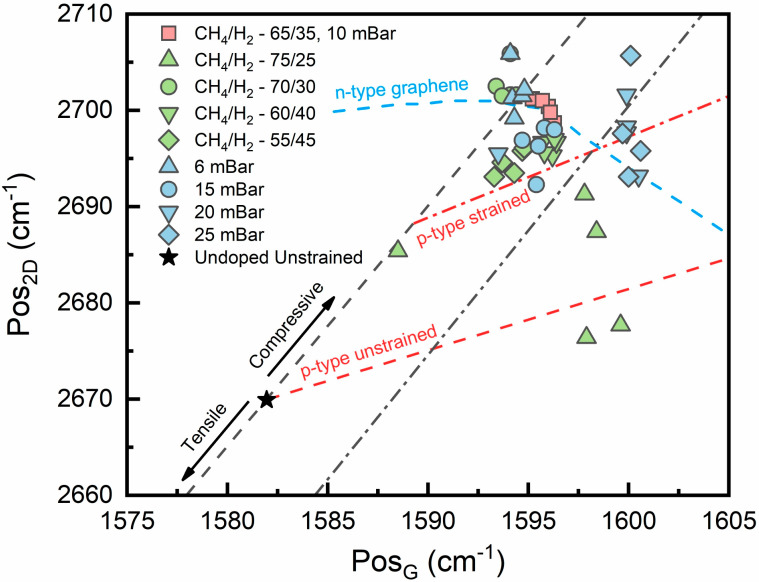
Pos_2D_ vs. Pos_G_ plot showing vector decomposition analysis of F and P sample sets. Conveniently, samples were given the same shapes and colors for distinction.

Furthermore, the effects of strain and doping were investigated using the Pos_2D_ vs. Pos_G_ plot (Figure 4) [62,65,66]. This established method suggests that most of our graphene samples are undoped or have a slight p-type doping. However, considering that most of our graphene samples are few-layered or thicker, these results may be inconclusive. Despite that, only several samples exhibit a higher Pos_G_ distribution. Vector decomposition analysis depicts the compressive strain present in most of our samples. Nevertheless, greater thickness might contribute to such observations, since the 2D peak position tends to blueshift with an increasing number of graphene layers [65,66].

**Figure 5:**
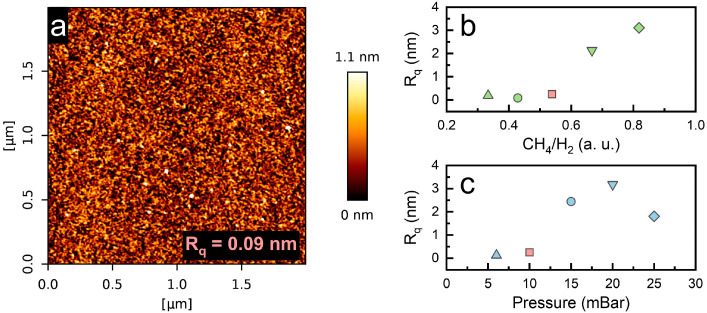
(**a**) Morphology of graphene synthesized using a CH_4_/H_2_ flow ratio of 35/65 and a pressure of 10 (R_q_ indicates the root mean square value of surface roughness), (**b**) R_q_ vs. gas-flow ratio plot, (**c**) R_q_ vs. pressure plot. The colors and shapes follow the same sample pattern.

**Figure 6:**
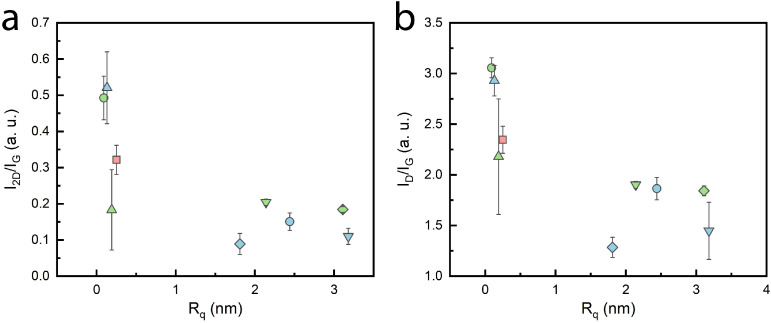
(**a**) Two-dimensional (2D) and G band ratio with respect to surface roughness, (**b**) plot showing correlation with surface roughness. The colors and shapes follow the same sample pattern.

The surface morphology of graphene synthesized on C-plane sapphire was studied with atomic force microscopy. Graphene structures are grainy, albeit smooth. The root mean square roughness (R_q_) of the samples was rather low, ranging from ~0.09 to 3.18 nm. The roughness of this size indicates the growth of planar graphene. The shared sample in both parameter sweeps had the lowest surface roughness (Figure 5a). It is important to note that the roughness of the pristine C-plane sapphire substrate was ~0.05 nm (Figure S3). The summarized roughness values according to CH_4_/H_2_ and pressure can be seen in Figure 5b,c, respectively. The produced graphene tends to become rougher with an increase in both the gas-flow ratio and pressure individually. Naturally, above certain gas-flow ratios and pressure threshold values, the surface roughness increases significantly. Low surface roughness was observed to correlate with higher I_2D_/I_G_ values; however, it also increased the I_D_/I_G_ ratio (Figure 6). Similar results have previously been observed with the synthesis of graphene performed on a Si(100) substrate [25]. It seems that the quality of graphene on the C-plane sapphire substrate is rather similar to the one that is grown on Si(100) substrate when using the MW-PECVD method. Temperatures well above 700 C might be required to achieve better quality graphene films on C-sapphire substrates, as reported by other studies [36,38]. Nevertheless, lower CH_4_ flow and lower pressure favor higher quality graphene synthesis. AFM images of the produced samples can be seen in Figures S4–S12.

**Figure 7:**
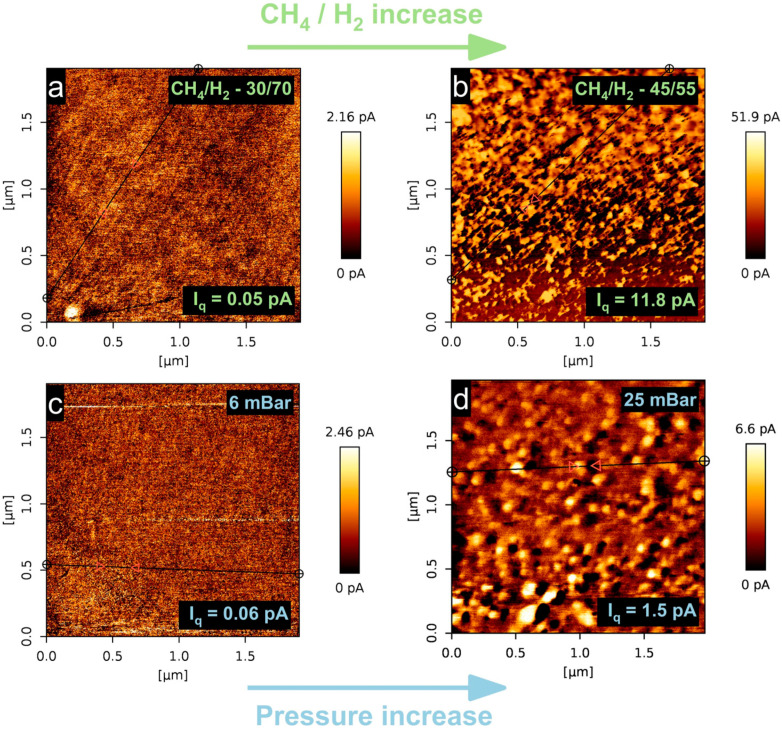
Conductive atomic force microscopy maps of four selected samples with varying synthesis conditions. (**a**) F2 sample, (**b**) F5 sample, (**c**) P1 sample, (**d**) P5 sample. I_q_ represents the root mean square of surface conductivity values.

**Figure 8:**
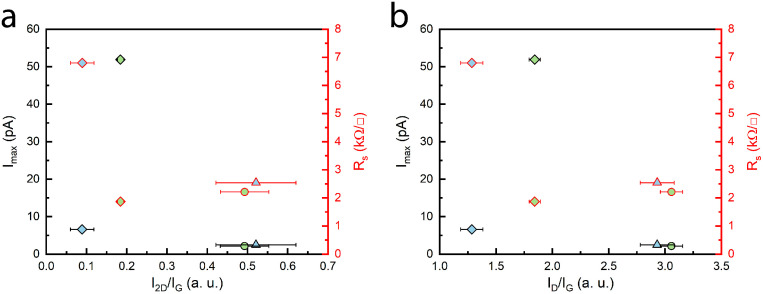
(**a**) Resistance characteristics vs. I_2D_/I_G_ plot showing conductance and sheet resistance variation with different thicknesses of graphene, (**b**) resistance characteristics vs. I_D_/I_G_ plot showing conductance and sheet resistance variation based on defects.

Conductive atomic force microscopy measurements were performed to observe the electrical properties of the graphene films. As mentioned in the experimental section, four samples were analyzed, bearing the highest and lowest I_2D_/I_G_ and I_D_/I_G_ ratios (Figure 7). Interestingly, most of the samples fell under the extremes of the chosen CH_4_/H_2_ gas-flow ratio and the experimental pressure sets. It seems that the growth conditions had a tremendous impact on the graphene current signal readouts. When the precursor gas-flow ratio was 30/70, the graphene exhibited the lowest current signals out of the four samples that were chosen, with a highest conductivity reaching 2.16 pA. When the gas-flow ratio was increased to 45/55, the current signal increased more than twentyfold, with the highest value being 51.9 pA. On the other hand, increasing the gas-flow ratio ultimately resulted in the thickest graphene sample. This would lead to the conclusion that we do not observe a monolayer, defect-free graphene effects when the thickness is low, knowing the highly conductive nature of pristine graphene [67]. The presence of graphene-like flakes or nanographene [68] could be observed and would explain the thickness-dependent increase in the current signal. We see a similar trend when pressure changes from 6 mBar to 25 mBar, where signal values change from 2.46 to 6.6 pA, respectively. It seems that the decrease in I_2D_/I_G_, seen in Figure 8a, is partly responsible for the increase in current signal. Similarly, the decrease in I_D_/I_G_ also increases the current signal. In principle, higher amounts of intrinsic grain boundary defects should not decrease the charge carrier mobility; however, it could indicate an extrinsic grain boundary effect in our films, or low-symmetry grain boundaries, consisting of tilts and angle changes between the grains, which changes the electronic properties dramatically [69].

The same four samples were further modified by synthesizing four, equally spaced, contacts, as mentioned in the experimental section, to determine the sheet resistance of the graphene films, using the four-point probe method. As seen in Figure 8, we can observe high R_s_ values when current signals acquired from conductive atomic force microscopy are low and rather low R_s_ values when current signals are high. In fact, the highest and lowest sheet resistance values were 6.8 kΩ/□ and 1.87 kΩ/□, respectively. The highest R_s_ value of the four was reported for the sample that was synthesized using a CH_4_/H_2_ ratio of 35/65 and a chamber pressure of 25 mBar. The lowest sheet resistance was observed when the sample was grown with a 45/55 gas-flow ratio and a chamber pressure of 10 mBar.

Our reported sheet resistance values are similar when compared to graphene synthesis on sapphire using other methods (see Table 2). It is evident that this growth method exhibits a lot of versality with respect to structure and resistance characteristics, when considering graphene synthesis on Al_2_O_3_ for sensor applications. Further improvements in this synthesis method are necessary to obtain higher quality graphene films. The variability that is introduced in the structure across a film could be mitigated when plasma mechanisms occurring in the chamber are thoroughly examined. To fully understand the effects of protective enclosures, different types of enclosure materials must also be investigated.

## 4. Conclusions

We have shown a novel approach to low-temperature, transfer-less graphene deposition that involves plasma-enhanced chemical vapor deposition. The quality of graphene can be varied by adjusting the pressure and the CH_4_/H_2_ gas-flow ratio, with I_D_/I_G_ values ranging from ~1 to ~4. The surface roughness of graphene mimics that of a substrate when low gas-flow ratios and chamber pressures are used. There is a notable reduction in the quality of graphene as a result of boundary defects and a grainy structure. Such a deposition evolves from poor nucleation. Despite the deposition of what appears to be a grainy multilayer graphene film, the electrical properties tend to be superior when compared to graphene synthesized using other CVD-based techniques. As a result, the sheet resistances can be as low as 1.87 kΩ/□. To further improve this synthesis method, a thorough investigation of plasma-associated effects must be considered. Naturally, plasma shielding is an important part of this synthesis technique, and the protective enclosure effect has to be investigated in more detail. By limiting the variability governed by these factors, the homogeneity of graphene films should be greatly improved, allowing for direct quality assessment based on the synthesis conditions used.

## Figures and Tables

**Table 1:** Synthesis conditions for investigated graphene/C-plane sapphire samples. Note that the sample F3P2 belongs to the F and P sets.

Set	Sample No.	Power, kW	H_2_, sccm	CH_4_, sccm	p, mBar	T, °C	t, min.
	F1	0.7	75	25	10	700	60
	F2	0.7	70	30	10	700	60
F	F3P2	0.7	65	35	10	700	60
	F4	0.7	60	40	10	700	60
	F5	0.7	55	45	10	700	60
	P1	0.7	65	35	6	700	60
	F3P2	0.7	65	35	10	700	60
P	P3	0.7	65	35	15	700	60
	P4	0.7	65	35	20	700	60
	P5	0.7	65	35	25	700	60
	S1	0.7	80	20	6	700	60
S	S2	0.7	75	25	6	700	60
	S3	0.7	75	25	25	700	60

**Table 2:** Sheet resistances measured for graphene on sapphire substrates.

Sample No.	Highest Surface Point, nm	Sheet Resistance, kΩ/□	Reference
A-plane sapphire	Low-pressure CVD	0.728	[29]
C-plane sapphire	Electron cyclotron resonance CVD	0.95	[39]
C-plane sapphire	High-temperature CVD	~1	[32]
Sapphire	Critical PECVD	4.1	[40]
C-plane sapphire	PECVD	1.87	This study

## Data Availability

No new data were created or analyzed in this study. Data sharing is not applicable to this article.

## Supplementary Materials

The following supporting information can be downloaded at: https://www.mdpi.com/article/10.3390/nano14201635/s1, Figure S1: Raw Raman spectra of C-plane sapphire and SiO_2_ for comparison; Figure S2: Plot showing a dominant defect type associated with graphene grown using different synthesis conditions: (a) I_D_/I_D’_ vs. CH_4_/H_2_ gas-flow ratio and (b) I_D_/I_D’_ vs. chamber pressure, based on fitted data using Raman theory [58,59]. Conveniently, samples were given the same shapes and colors for distinction, as in the main text.; Figure S3: AFM image of C-plane sapphire substrate; Figure S4: AFM image of F1 sample; Figure S5: AFM image of F2 sample; Figure S6: AFM image of F3P2 sample; Figure S7: AFM image of F4 sample; Figure S8: AFM image of F5 sample; Figure S9: AFM image of P1 sample; Figure S10: AFM image of P3 sample; Figure S11: AFM image of P4 sample; Figure S12: AFM image of P5 sample.
