# Supplementary material for: Influence of Synthesis Parameters on Structure and Characteristics of the Graphene Grown Using PECVD on Sapphire Substrate
Source: Nanomaterials (Basel). 2024 Oct 12;14(20):1635. doi: 10.3390/nano14201635 (PMC11509920; doi:10.3390/nano14201635)
Supplement: Supplementary file 1 [file nanomaterials-14-01635-s001.zip › nanomaterials-3227035-supplementary.pdf]

nanomaterials

Supplementary Materials

Influence of Synthesis Parameters on Structure and Characteristics of the Graphene Grown Using PECVD on Sapphire Substrate

Šarūnas Jankauskas [1], Šarūnas Meškinis [1], Nerija Žurauskienė [2] and Asta Guobienė [1,*]

[1] Institute of Materials Science, Kaunas University of Technology, K. Baršausko St. 59, LT-51423 Kaunas, Lithuania; sarunas.jankauskas@ktu.lt (Š.J.) sarunas.meskinis@ktu.lt (Š.M.)
[2] Department of Functional Materials and Electronics, Center for Physical Sciences and Technology, Saulėtekio av. 3, LT-10257 Vilnius, Lithuania; nerija.zurauskiene@ftmc.lt
* Correspondence: asta.guobiene@ktu.lt

Supplement S1. Additional information acquired with Raman spectroscopy

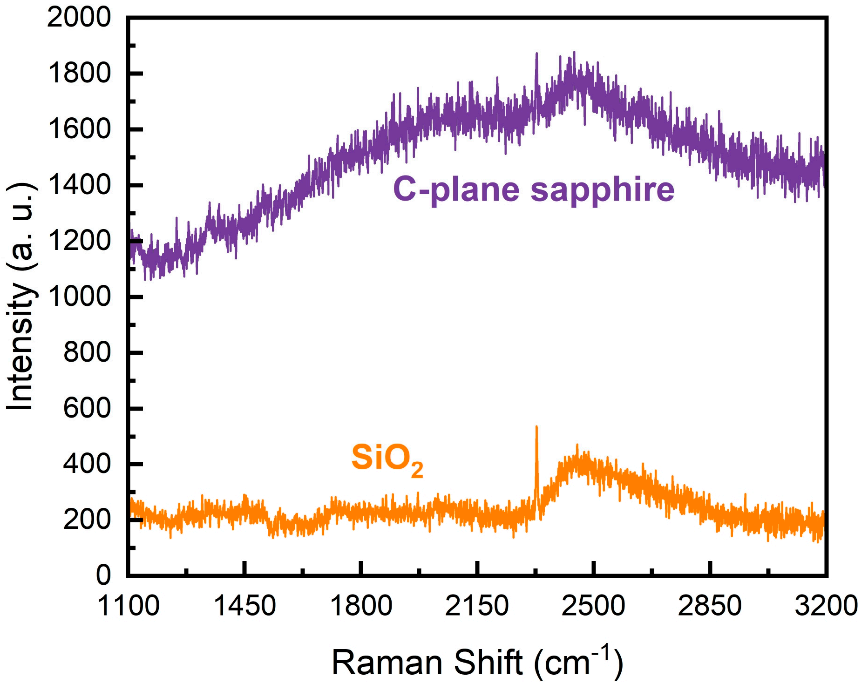

Figure S1. Raw Raman spectra of C-plane sapphire and SiO_2 for comparison.

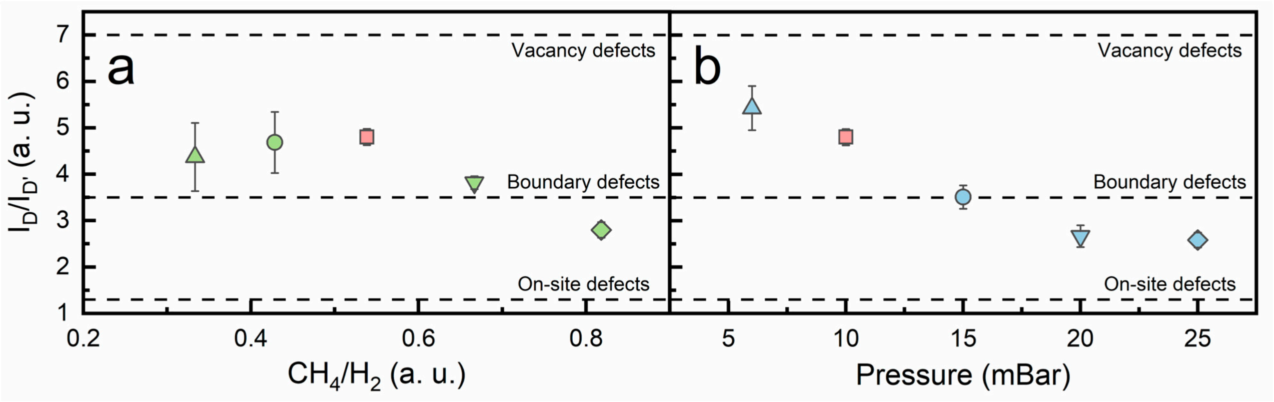

Figure S2. Plot showing a dominant defect type associated with graphene grown using different synthesis conditions. (a) $I_D/I_{D'}$ vs. CH_4/H_2 gas flow ratio and (b) $I_D/I_{D'}$ vs. chamber pressure used, based on fitted data using Raman theory [1,2]. Conveniently, samples were given the same shapes and colors for distinction as in the main text.

Supplement S2. AFM measurements

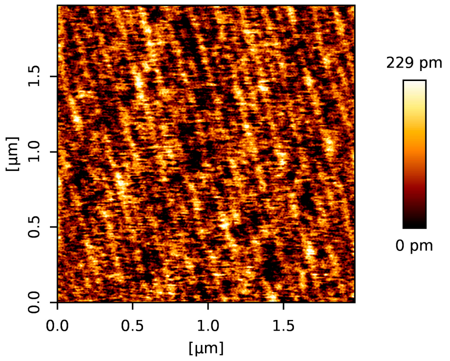

Figure S3. AFM image of C-plane sapphire substrate.

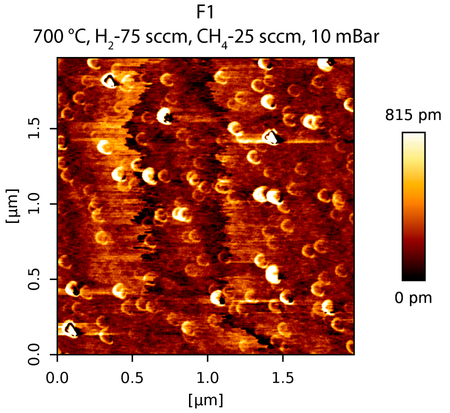

Figure S4. AFM image of F1 sample.

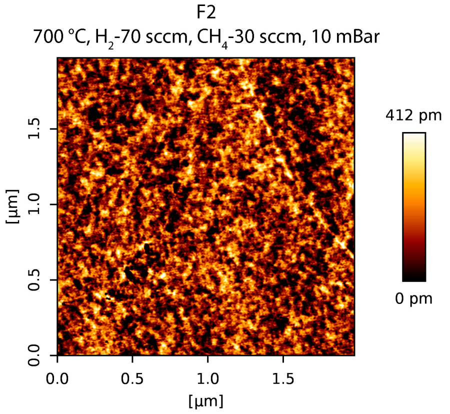

Figure S5. AFM image of F2 sample.

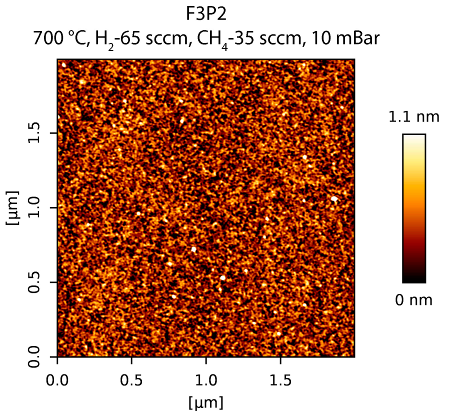

Figure S6. AFM image of F3P2 sample.

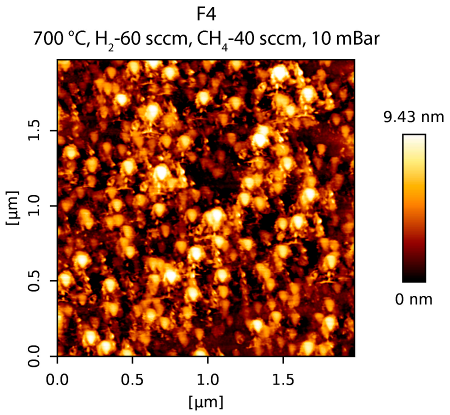

Figure S7. AFM image of F4 sample.

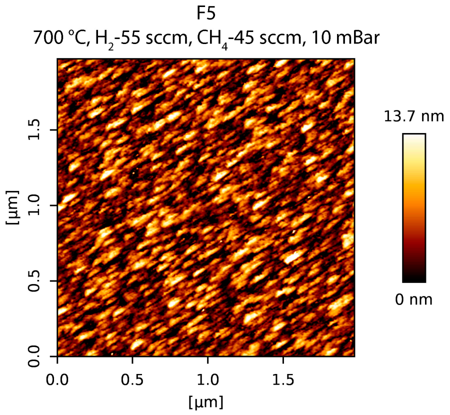

Figure S8. AFM image of F5 sample.

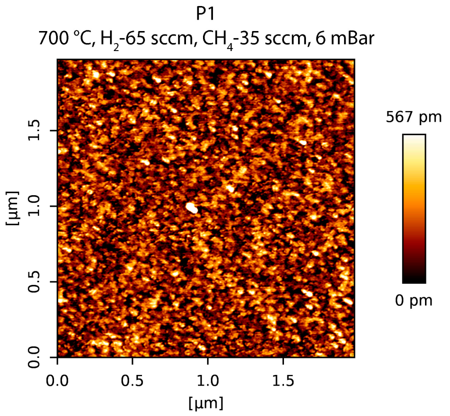

Figure S9. AFM image of P1 sample.

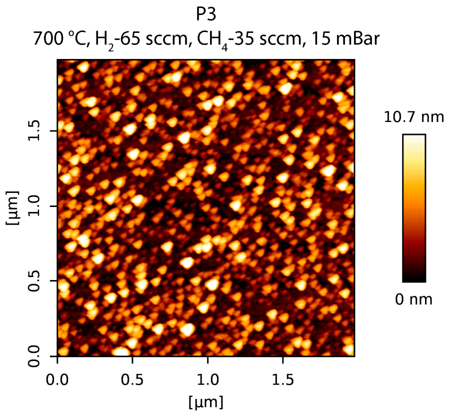

Figure S10. AFM image of P3 sample.

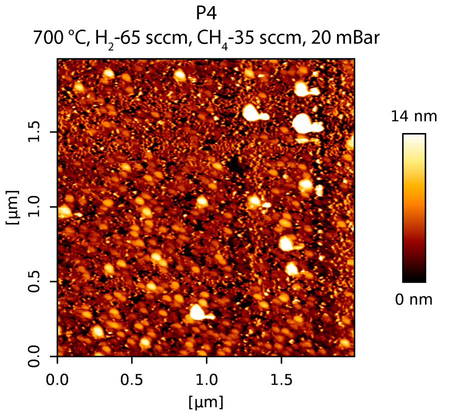

Figure S11. AFM image of P4 sample.

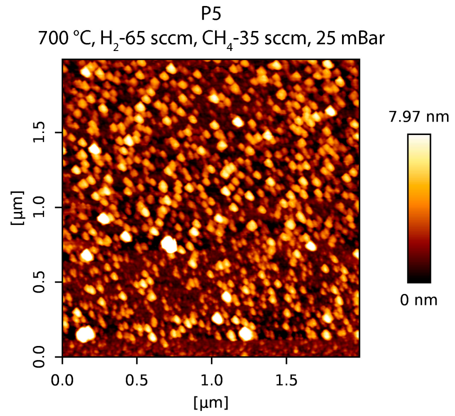

Figure S12. AFM image of P5 sample.

References

1. Eckmann, A.; Felten, A.; Mishchenko, A.; Britnell, L.; Krupke, R.; Novoselov, K.S.; Casiraghi, C. Probing the Nature of Defects in Graphene by Raman Spectroscopy. *Nano Lett* **2012**, *12*, 3925–3930, doi:10.1021/nl300901a.
2. Venezuela, P.; Lazzeri, M.; Mauri, F. Theory of Double-Resonant Raman Spectra in Graphene: Intensity and Line Shape of Defect-Induced and Two-Phonon Bands. *Phys Rev B* **2011**, *84*, 035433, doi:10.1103/PhysRevB.84.035433.
